# Evaluating the Shock Index, Revised Assessment of Bleeding and Transfusion (RABT), Assessment of Blood Consumption (ABC) and novel PTTrauma score to predict critical transfusion threshold (CAT) in penetrating thoracic trauma

**DOI:** 10.1038/s41598-024-62579-x

**Published:** 2024-06-11

**Authors:** Mario Miguel Barbosa Rengifo, Alberto F. Garcia, Adolfo Gonzalez-Hada, Nancy J. Mejia

**Affiliations:** 1 Department of Surgery, Universidad del Valle, Cl. 4B #36-00, El Sindicato, Cali Valle del Cauca, Cali, Colombia; 2 Department of Surgery and Clinical Research Center, Fundación Valle del Lili, Cali, Colombia; 3 Universidad Icesi, Facultad de Ciencias de la Salud, Cali, Colombia

**Keywords:** Thoracic injuries, Blood transfusion, Hemothorax, Pneumothorax, Cardiac tamponade, General surgery, Epidemiology, Outcomes research, Diagnosis, Prognosis, Public health, Health care, Medical research, Signs and symptoms

## Abstract

The shock index (SI) has been associated with predicting transfusion needs in trauma patients. However, its utility in penetrating thoracic trauma (PTTrauma) for predicting the Critical Administration Threshold (CAT) has not been well-studied. This study aimed to evaluate the prognostic value of SI in predicting CAT in PTTrauma patients and compare its performance with the Assessment of Blood Consumption (ABC) and Revised Assessment of Bleeding and Transfusion (RABT) scores. We conducted a prognostic type 2, single-center retrospective observational cohort study on patients with PTTrauma and an Injury Severity Score (ISS) > 9. The primary exposure was SI at admission, and the primary outcome was CAT. Logistic regression and decision curve analysis were used to assess the predictive performance of SI and the PTTrauma score, a novel model incorporating clinical variables. Of the 620 participants, 53 (8.5%) had more than one CAT. An SI > 0.9 was associated with CAT (adjusted OR 4.89, 95% CI 1.64–14.60). The PTTrauma score outperformed SI, ABC, and RABT scores in predicting CAT (AUC 0.867, 95% CI 0.826–0.908). SI is a valuable predictor of CAT in PTTrauma patients. The novel PTTrauma score demonstrates superior performance compared to existing scores, highlighting the importance of developing targeted predictive models for specific injury patterns. These findings can guide clinical decision-making and resource allocation in the management of PTTrauma.

## Introduction

Penetrating thoracic trauma (PTTrauma) represents a significant challenge in trauma services^1–3^. In Cali, thoracic trauma was observed in 45% of critically attended trauma patients and accounted for 52% of preventable trauma-related deaths^4,5^. The mortality rates associated with thoracic trauma stand at 30%, but in cases of cardiac trauma, these rates can escalate to 90% if appropriate care is not provided^2,3^. The Advanced Trauma Life Support protocol aims to diagnose and address immediately life-threatening injuries in thoracic trauma, such as airway obstruction, tension pneumothorax, pneumothorax, massive hemothorax, flail chest, and cardiac tamponade^2^. In cases of PTTrauma, a cascade of events, including vascular lesions, hemorrhage, and exsanguination, occurs, especially in the context of massive hemothorax and cardiac tamponade^6^. Early diagnosis of hemorrhage, prevention of hemodilution, treatment of coagulopathy, surgical repair, Focused Assessment with Sonography in Trauma (FAST) ultrasonography, restrictive infusion of crystalloids, administration of tranexamic acid, and damage control surgery are some of the crucial interventions in damage control resuscitation for these injuries^7–9^.

The management of penetrating trauma involves the diagnosis, treatment, and prevention of coagulopathy, which includes laboratory tests, predictive scores, the transfusion of blood components, and the prophylactic administration of tranexamic acid^8,10–12^. The concept of massive transfusion (MT) has evolved from the classical definition of transfusing 10 units of packed red blood cells (PRBCs) over 24 h to transfusing 10 units of PRBCs in 6 h. More recent concepts involve the speed of transfusion, such as the critical administration threshold (CAT), which is defined by the number of times that a total of 3 PRBCs transfused within an hour. CAT + patients are quantified by the number of times CAT + was reached, that is, once (CAT1), twice (CAT2), three times (CAT3), or 4 or more times (CAT4)^13–16^. Predictive scoring has also advanced from simpler models incorporating few variables and less complexity, such as the Assessment of Blood Consumption (ABC) model that uses heart rate, systolic pressure, FAST, and penetrating mechanism of trauma^17^; to more complex models with additional variables, like the Trauma Associated Severe Hemorrhage (TASH) that incorporates base deficit and large bone fractures alongside ABC score variables^18^. However, the need for laboratory tests and ultrasonography limit its application in low-resource scenarios^19,20^.

For this purpose, the shock index (SI) was revisited. It is defined as the ratio of heart rate (HR) to systolic blood pressure (SBP) and is considered normal within the 0.5–0.7 range^21^. Previous studies have demonstrated the association between the SI and the need for massive transfusion in unselected trauma patients. However, a knowledge gap persists due to the low prevalence of penetrating trauma and the lack of specificity regarding the infrequent occurrence of PTTrauma in these studies^22,23^. The Revised Assessment of Bleeding and Transfusion (RABT) Score improves upon previous methods by replacing hypotension and tachycardia cutoff points with an SI greater than 1.0 and incorporating pelvic fractures into the scoring system. This modification significantly enhances its predictive accuracy for massive transfusion. However, the score does not account for thoracic injury location in its assessment^24,25^. Further studies associated SI with mortality, the need for surgery but not with CAT in the context of PTTrauma^26,27^.

Despite the existence of various predictive scores for massive transfusion, there is a lack of studies specifically focusing on PTTrauma. The unique physiological challenges and the high risk of life-threatening hemorrhage associated with this injury pattern warrant the development of a more targeted predictive model. This study aims to address this gap by evaluating the discriminative capacity of the SI in predicting the need for massive transfusion, as measured by the CAT, in patients with PTTrauma. Furthermore, we compare the performance of the SI with existing predictive scores, namely the ABC and RABT scores, to determine its utility in this specific context.

## Methods

### Study design and data

We conducted a type 2 prognostic factor retrospective close cohort study^28,29^. The study utilized data from an already published study that recruited patients in the year 2016^30^, as well as blood transfusion data from electronic records. The institutional review board exempted the study from informed consent requirements. This exemption was granted by the institutional review board due to the immediate and serious risk to patient integrity presented by PTTrauma, this is because PTTrauma is a life-threatening situation that demands urgent action, and often, the patient's clinical condition makes it impractical to secure authorization swiftly. Consequently, this study solely used de-identified data from blood bank database, and was also approved by the institutional review board. The study included patients aged 14 years and above who were admitted to the Hospital Universitario del Valle in Cali, Colombia, with penetrating thorax trauma in the year 2016. Patients with an Injury Severity Score (ISS) below 9, suspected pregnancy, spinal cord injury trauma in cervical and thoracic dermatomes below fourth thoracic vertebra were excluded^31^. The study collected data that included age, mechanism of injury, vital signs, topographic location of injuries, physical examination findings, Abbreviated Injury Score (AIS), ISS, Glasgow Coma Scale score, Focused Assessment with Sonography for Trauma (FAST), and surgical procedures. Additionally, the study extracted data on transfused PRBCs, plasma, platelets, and cryoprecipitate from the blood bank database. The study used the CAT as the outcome variable, which is defined as the transfusion of more than 3 units of PRBCs within 1 hour^15^. The SI was the primary exposure and predictor variable, calculated from the vital signs at admission by dividing the heart rate by the systolic blood pressure. Additionally, a SI cutoff of 0.9 was selected based on previous studies, and its appropriateness was evaluated through statistical analysis^21,26^. The study adhered to the PROGRESS Framework for Prognostic Studies, Transparent Reporting of a Multivariable Prediction Model for Individual Prognosis or Diagnosis (TRIPOD), and the Strengthening the Reporting of Observational Studies in Epidemiology (STROBE) guidelines^29,32,33^.

### Statistical analysis

The study reported baseline, outcome, and demographic characteristics using descriptive statistics. For categorical variables, the Chi-Square test was applied if n was greater than or equal to 5, and Fisher’s exact test was used otherwise. For continuous variables, the Student’s t-test was employed if the assumption of normality held or if n was greater than 30, and the Wilcoxon rank sum test was used otherwise. The Youden's index helped determine the optimal univariate SI cutoff value, and comparisons were made with previous studies^34^. Predictors underwent inclusion in multiple logistic regression with backward variable elimination through stepwise selection (a selection threshold of p ≤ 0.20 minimized residual confounding) considering only variables that were available to the trauma team before the outcome. The study calculated adjusted odds ratios (OR), likelihood ratios, and calibration evaluated by the Hosmer–Lemeshow goodness-of-fit test and pseudo-R-squared. The study considered a p-value of less than 0.05 to be significant. Discrimination measures such as the Area Under the Curve (AUC), sensitivity, specificity, positive likelihood ratios (LR +), and negative likelihood ratios (LR-) were calculated. AUROCs were compared using Delong’s test. Score performance was also assessed using decision curve analysis (DCA)^35^. The DCA plots the net benefit of each score against the threshold probability, which represents the minimum probability of the outcome at which a clinician would consider intervention^36^. A higher net benefit indicates that the score more accurately identifies patients who will benefit from the intervention while minimizing the number of patients unnecessarily treated.^37,37^. The net intervention avoided plot was also generated, this plot ilustrate the number of interventions (i.e., massive transfusions) avoided per 100 patients across different threshold probabilities. Sample size calculations relied on the methods of Hsieh^38^ for logistic regression and Obuchowski for the Area Under the Curve^39^. With these methods, a difference of 10% between groups and an AUC of 0.8 could be detected at 80% power (α = 0.05) with a minimum of 50 patients with CAT and 200 without CAT. The study used R statistical software version 4.2.1 for analysis.

### Ethical aspects

The study received approval from the institutional review board of Universidad del Valle and Hospital Universitario del Valle (approval no: 012-020) and adhered to the Helsinki Declaration 2013 and Technical and Scientific Regulations for healthcare research established in Colombia under Resolution 8430. It was classified as without risk. The research was granted an exemption from the informed consent requirement by the Universidad del Valle and Hospital Universitario del Valle (approval no: 012-020) requirement based on the immediate and serious risk posed by PTTrauma to patient integrity. PTTrauma, being a life-threatening condition requiring urgent action, often made obtaining swift authorization impractical due to the patient's clinical condition^40^. Furthermore, data anonymity was rigorously maintained throughout the study to protect participant privacy, following the guidelines of the Health Insurance Portability and Accountability Act (HIPAA) Privacy Rule. All human participants' names and other HIPAA identifiers were systematically removed from all sections of the manuscript^41^. The study did not include participants below the age of 18, in accordance with the definition of minors as per Colombian law.

## Results

**Figure 1:**
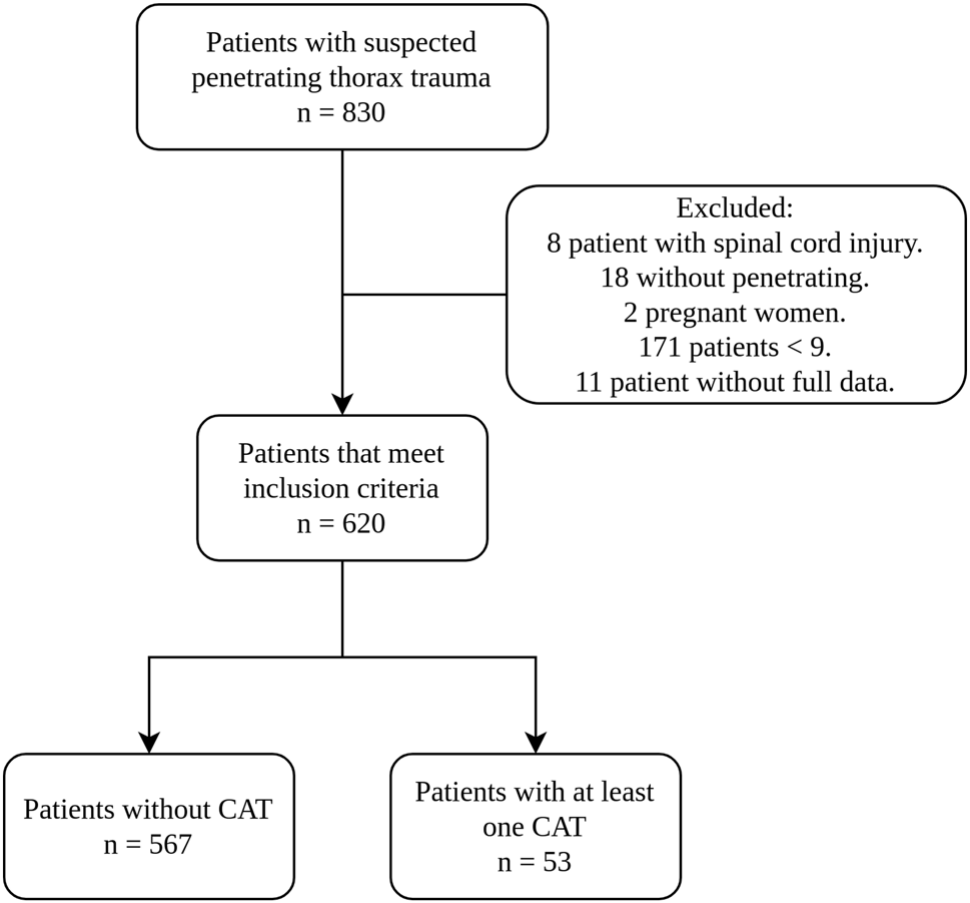
Patient inclusion flowchart: selection and exclusion criteria.

During the study period, 830 patients with initially suspected penetrating thoracic trauma were entered into the registry of this 620 patients met the inclusion criteria (Fig. 1). Demographic data showed that the majority of patients (585 out of 620, or 94.4%) were men, with a median age of 25.0 years (interquartile range [IQR] 20–32 years). CAT was met in 8.5% of cases (53 out of 620). Stabbing was the most frequent mechanism of injury, accounting for 62.7% of cases (389 out of 620), followed by gunshot wounds at 37.3% (231 out of 620). One in three patients had a lesion in the precordial, pure thoracic anterior, or posterior topographic location. Hypoventilation was the most frequent physical finding, present in 68.2% of patients (423 out of 620). More than half of the patients had hemothorax and pneumothorax. Surgery was performed within 4 hours of admission in 18.4% of cases (114 out of 620), vascular thoracic lesions were present in 7.9% (49 out of 620), and the overall mortality was 5% (31 out of 620). PRBCs were transfused in 23.4% of patients (142 out of 620), fresh frozen plasma in 10.9% (64 out of 620), platelets in 5.4% (30 out of 620), and cryoprecipitate in 1.6% (6 out of 620) (Tables 1, 2).

**Table 1:** Baseline characteristics stratified by shock index category at admission.

Variables	SI < 0.9	SI > 0.9	Cohort	p value
	N = 469	N = 151		
CAT > 1 within 4 hours from admission	15 (3.2)	38 (25.2)	53 (8.5)	< 0.001^c^
Age (years), median (IQR)^a^	25.0 (20–32)	26.0 (19–34)	25.0 (20–32)	0.89^b^
Sex				0.32^c^
Women, n (%)	24 (5.1)	11 (7.3)	35 (5.6)	
Men, n (%)	445 (94.9)	140 (92.7)	585 (94.4)	
Vital signs				
Systolic blood pressure (mmhg), median (IQR)^a^	120.0 (110–131)	90.0 (80–100)	110.0 (100–129)	< 0.001^b^
Diastolic blood pressure (mmhg), median (IQR)^a^	72.0 (69–80)	60.0 (50–70)	70.0 (60–80)	< 0.001^b^
Heart ratey (beats per minute), median (IQR)^a^	80.0 (72–88)	110.0 (98–120)	84.0 (75–98)	< 0.001^b^
Respiratory rate (breaths per minute), median (IQR)^a^	20 (18–22)	22 (20–24)	20 (18–22)	< 0.001^b^
ISS, median (IQR)^a^	11.0 (10–15)	17.0 (11–26)	12.0 (10–17)	< 0.001^b^
Glasgow Coma Scale equal to 15, n (%)	423 (90.2)	100 (66.2)	523 (84.4)	< 0.001^b^
Mechanism of trauma				0.81^c^
Stabbing, n (%)	293 (62.5)	96 (63.6)	389 (62.7)	
Gunshot, n (%)	176 (37.5)	55 (36.4)	231 (37.3)	
Topography of lesion, n (%)				
Precordial	116 (24.7)	62 (41.1)	178 (28.7)	< 0.001^c^
Pure Thoracic anterior	135 (28.8)	67 (44.4)	202 (32.6)	< 0.001^c^
Pure Thoracic posterior	177 (37.7)	59 (39.1)	236 (38.1)	0.77^c^
Epigastric	16 (3.4)	8 (5.3)	24 (3.9)	0.3^c^
Transmediastinal	28 (6.0)	16 (10.6)	44 (7.1)	0.05^c^
Thoracic operculum	68 (14.5)	19 (12.6)	87 (14.0)	0.56^c^
Physical examination findings, n (%)				
Jugular engorgement	1 (0.2)	4 (2.6)	5 (0.8)	0.01^c^
Muffled heart sounds	10 (2.1)	9 (6.0)	19 (3.1)	0.03^c^
Hypoventilation	310 (66.1)	113 (74.8)	423 (68.2)	0.04^c^
Hypertympany	6 (1.3)	3 (2.0)	9 (1.5)	0.46^c^
Thoracic asymmetry	39 (8.3)	21 (13.9)	60 (9.7)	0.04^c^
Blowing wound	64 (13.6)	25 (16.6)	89 (14.4)	0.38^c^
Subcutaneous emphysema	86 (18.3)	12 (7.9)	98 (15.8)	0.002^c^
Transient hypotension	67 (14.3)	83 (55.0)	150 (24.2)	< 0.001^c^
Cardiac tamponade signs	40 (8.5)	75 (49.7)	115 (18.5)	< 0.001^c^
Respiratory distress	65 (13.9)	57 (37.7)	122 (19.7)	< 0.001^c^
Pelvic fracture	0 (0)	0 (0)	0 (0)	< 0.001^c^
Patient without FAST ultrasound, n(%)	41 (8.7)	19 (12.6)	60 (9.7)	0.17^c^
Positive FAST ultrasound	428 (91.3)	132 (87.4)	560 (90.3)	< 0.001^c^
Chest tube insertion in emergency room	317 (67.6)	119 (78.8)	436 (70.3)	0.009^c^
Hemothorax, n (%)				< 0.001^c^
Unilateral	237 (50.5)	92 (60.9)	329 (53.1)	
Bilateral	19 (4.1)	15 (9.9)	34 (5.5)	
Pneumothorax, n (%)				0.003^c^
Unilateral	209 (44.6)	50 (33.1)	259 (41.8)	
Bilateral	16 (3.4)	12 (7.9)	28 (4.5)	
Surgery, n (%)				
Surgery < 4 h from admission	52 (11.1)	62 (41.1)	114 (18.4)	0.001^c^
Outcome of surgery, n(%)				0.002^c^
Negative	301 (64.2)	69 (45.7)	370 (59.7)	
No Therapeutic	19 (4.1)	7 (4.6)	26 (4.2)	
Therapeutic	149 (31.8)	75 (49.7)	224 (36.1)	
Pericardic window, n (%)				< 0.001^c^
Not done	356 (75.9)	83 (55.0)	439 (70.8)	
Yes, negative	91 (19.4)	45 (29.8)	136 (21.9)	
Yes, positive	22 (4.7)	23 (15.2)	45 (7.3)	
Cardiac Tamponade, n (%)	19 (4.1)	19 (12.6)	38 (6.1)	< 0.001^c^
Thoracic vascular lesion, n (%)	21 (4.5)	28 (18.5)	49 (7.9)	< 0.001^c^
Surgical lung intervention, n(%)				
Thorax Drainage	313 (66.7)	88 (58.3)	401 (64.7)	0.1^c^
Segmental resection or tractotomy	5 (1.1)	10 (6.6)	15 (2.4)	< 0.001^c^
Lobectomy	3 (0.6)	2 (1.3)	5 (0.8)	0.6^c^
Pneumoraphy	4 (0.9)	16 (10.6)	20 (3.2)	< 0.001^c^
Without lesion	144 (30.7)	35 (23.2)	179 (28.9)	< 0.001^c^
Cardiac lesion, n (%)				0.001^c^
Pericardic	11 (2.3)	15 (9.9)	26 (4.2)	
Heart	10 (2.1)	5 (3.3)	15 (2.4)	
Without lesion	448 (95.5)	131 (86.8)	579 (93.4)	
Damage control surgery, n (%)				< 0.001^c^
Abdominal	8 (1.7)	6 (4.0)	14 (2.3)	
Thoracic	9 (1.9)	16 (10.6)	25 (4.0)	
Thoracic y abdominal	4 (0.9)	3 (2.0)	7 (1.1)	
Not done	448 (95.5)	126 (83.4)	574 (92.6)	
Type of surgery, n (%)				
Laparotomy	82 (17.5)	47 (31.1)	129 (20.8)	< 0.001^c^
Thoracoscopy	59 (12.6)	19 (12.6)	78 (12.6)	1^c^
Thoracotomy	31 (6.6)	41 (27.2)	72 (11.6)	< 0.001^c^
Not done	243 (51.8)	37 (24.5)	280 (45.2)	< 0.001^c^
Mortality, n (%)	13 (2.8)	18 (11.9)	31 (5.0)	< 0.001^c^
Cause of death, n (%)				0.31^c^
Coagulophaty	2 (16.7)	2 (10.5)	4 (12.9)	
Multiorganic dysfunction	2 (16.7)	5 (26.3)	7 (22.6)	
Exanguination	4 (33.3)	1 (5.3)	5 (16.1)	
Refractary hypovolemic shock	4 (33.3)	11 (57.9)	15 (48.4)	
Method of lesion identification, n (%)				
Evolution and Echocardiography	313 (66.7)	83 (55.0)	396 (63.9)	< 0.001^c^
Echocardiography	17 (3.6)	0 (0.0)	17 (2.7)	
Clinical evolution	47 (10.0)	10 (6.6)	57 (9.2)	
Thorax surgery	92 (19.6)	58 (38.4)	150 (24.2)	

^a^Shapiro–Wilk test < 0.05.

^b^Mann–Whitney U test

^c^Fisher exact test

**Table 2:** Transfusion of Blood components in the cohort.

Number of transfused hemocomponents	PRBCs, n (%)	Fresh frozen Plasma, n (%)	Platelets, n (%)	Cryoprecipitate, n (%)
0	478 (76.6)	556 (89.1)	590 (94.6)	614 (98.4)
1	20 (3.2)	2 (0.3)	26 (4.2)	1 (0.2)
2	35 (5.6)	13 (2.1)	6 (1.0)	0 (0.0)
3	26 (4.2)	6 (1.0)	1 (0.2)	0 (0.0)
4	16 (2.6)	18 (2.9)	0 (0.0)	2 (0.3)
5	13 (2.1)	2 (0.3)	1 (0.2)	0 (0.0)
> 5	36 (5.8)	27 (4.3)	0 (0.0)	7 (1.1)

**Figure 2:**
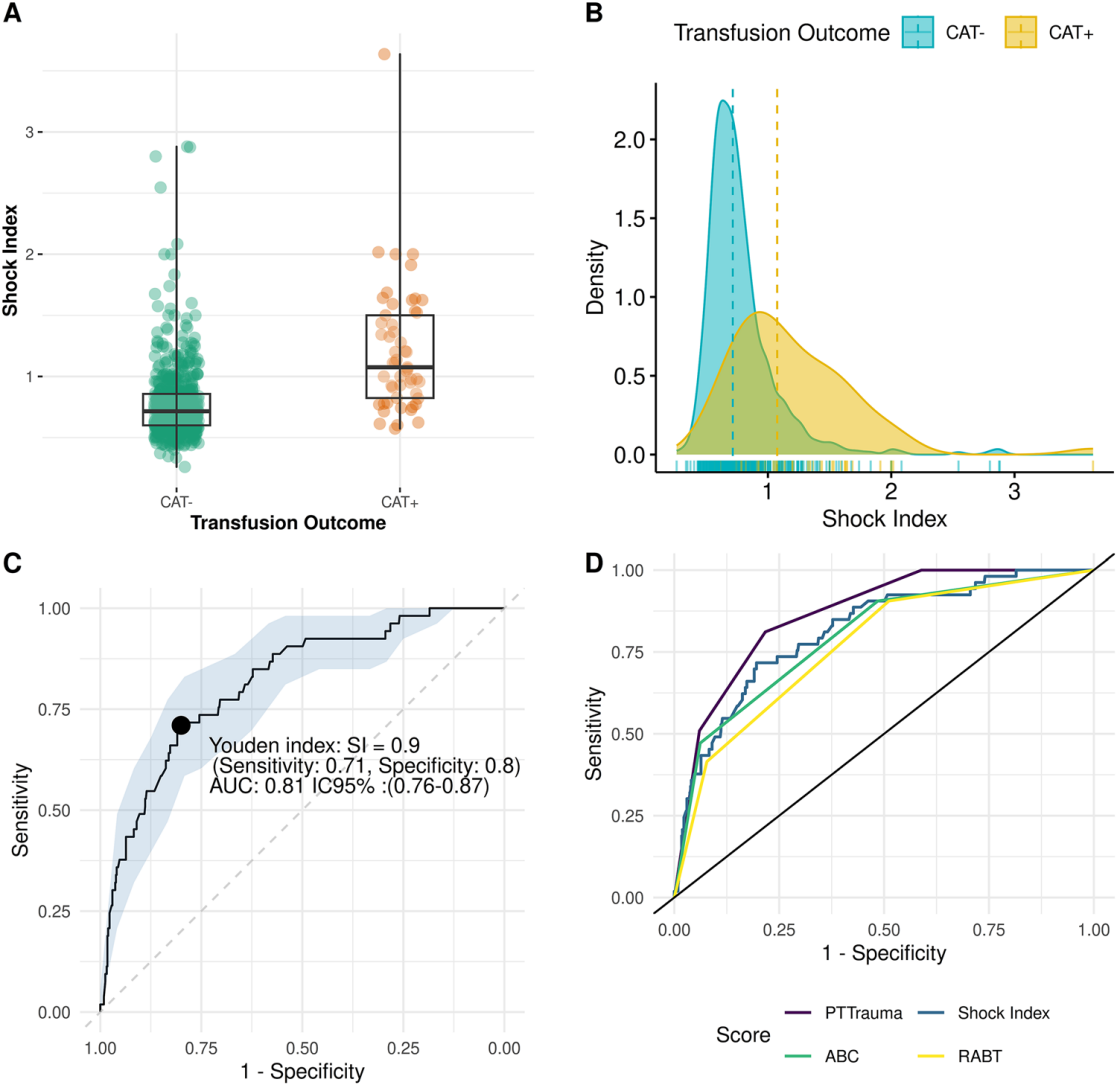
Operating characteristics of Shock Index and PTTrauma model for the prediction of CAT > 1 within 4 h from admission.

The univariate analysis revealed a non-normal distribution of SI among the groups, with a clear distinction between the CAT+ and CAT− groups (Fig. 2A,B). The SI demonstrated similar performance to the RABT and ABC scores in predicting transfusion outcomes. The maximum Youden's index identified the SI cutoff of > 0.9 as the most favorable value (Fig. 2C). Based on this finding, the SI variable was dichotomized using the cutoff value of 0.9 for subsequent analyses.

The SI > 0.9, transient hypotension, cardiac tamponade signs, surgery within 4 h of admission, and positive FAST Ultrasound were found to be independently correlated with CAT. We assessed an interaction term between the SI > 0.9 and surgery within 4 h of admission, but it was not correlated with our outcome in the final model PTTrauma Score (Table 3). The SI > 0.9 yielded a crude OR of 10.16 (95% CI 5.40, 19.10) and an adjusted OR of 4.89 (95% CI 1.64, 14.60) for PRCB transfusion beyond the CAT definition. The Hosmer–Lemeshow test statistic indicated an acceptable model fit (χ^2^ = 11.309, df = 8, p = 0.2). In the final model, the presence of ≥ 2 variables (SI > 0.9, transient hypotension, sings of cardiac tamponade, positive FAST Ultrasound) was selected. We chose these variables due to their availability to the low-complexity healthcare trauma team before the outcome occurred (Table 3).

**Table 3:** Logistic regression model to adjust the prognostic capacity of the Shock index.

	Crude		Adjusted	
	OR (95% CI)	p value	OR (95% CI)	p value
Intercept^a,b^	–	–	–	< 0.001
SI > 0.9^a,b^	10.16 (5.40, 19.10)	< 0.001	4.89 (1.64, 14.60)	0.01
Transient hypotension and cardiac tamponade signs^a,b^	18.18 (8.64, 38.28)	< 0.001	4.93 (2.08, 11.66)	< 0.001
Surgery < 4 hours from admission^a,b^	14.72 (7.81, 27.73)	< 0.001	8.61 (2.73, 27.18)	< 0.001
Positive FAST ultrasound^a,b^	4.09 (2.11, 7.95)	< 0.001	2.81 (1.34, 5.91)	0.01
Interaction term SI > 0.9 and surgery < 4 h from admission^a,b^	–	–	0.40 (0.10, 1.66)	0.21

^a^Hosmer–Lemeshow Test, p = 0.2

^b^pseudo-R2 = 0.35

The SI demonstrated good discriminative capacity, followed closely by ABC and RABT. Notably, the PTTrauma Score outperformed all three scores, showing superiority over SI, ABC, and RABT. (Table 4, Fig. 2D) The SI > 0.9, yields a sensitivity of 71.7% and a specificity of 80.0%. The PTTrauma score, at a cutoff of 2, achieves a sensitivity of 81% and a specificity of 78%. The ABC score, also at a cutoff of 2, has a sensitivity of 91% but a lower specificity of 51%. Similarly, the RABT score, at a cutoff of 2, has a sensitivity of 91% and a specificity of 49% (Table 5).

**Table 4:** Comparison of dicriminative power among Shock index, RABT and ABC score.

Score	AUC (95% CI)
SI (Univariate)	0.815 (0.755–0.874)
ABC	0.797 (0.738–0.856)
RABT	0.768 (0.709–0.827)
PTTrauma score^a^	0.867 (0.826–0.908)

^a^The Delong’s test yielded a p-value < 0.05 when compared with the SI, ABC, and RABT scores with PTTrauma Score.

**Table 5:** Comparison of operating characteristics and optimal cutoffs among Shock Index, RABT, and ABC scores.

Score^a^	Cutoff	Depth	True Positive	False Positive	True Negative	False Negative	Accuracy	Misclassification rate	Sensitivity	Specificity	Precision	Positive predictive value	Negative predictive value	Positive likelihood ratio	Negative likelihood ratio
Shock Index	**0.908**	**0.2435**	**38**	**113**	**454**	**15**	**0.7935**	**0.2065**	**0.7170**	**0.8007**	**0.2517**	**0.2517**	**0.9680**	**3.5976**	**0.3535**
PTTrauma	Inf	0.00	0	0	567	53	0.91	0.09	0.00	1.00	–	–	0.91	–	1.00
	3	0.10	27	34	533	26	0.90	0.10	0.51	0.94	0.44	0.44	0.95	8.50	0.52
	**2**	**0.27**	**43**	**123**	**444**	**10**	**0.79**	**0.21**	**0.81**	**0.78**	**0.26**	**0.26**	**0.98**	**3.74**	**0.24**
	1	0.62	53	334	233	0	0.46	0.54	1.00	0.41	0.14	0.14	1.00	1.70	0.00
	0	1.00	53	567	0	0	0.09	0.91	1.00	0.00	0.09	0.09	–	1.00	–
ABC	Inf	0.00	0	0	567	53	0.91	0.09	0.00	1.00	–	–	0.91	–	1.00
	4	0.02	4	6	561	49	0.91	0.09	0.08	0.99	0.40	0.40	0.92	7.13	0.93
	3	0.10	25	35	532	28	0.90	0.10	0.47	0.94	0.42	0.42	0.95	7.64	0.56
	**2**	**0.52**	**48**	**275**	**292**	**5**	**0.55**	**0.45**	**0.91**	**0.51**	**0.15**	**0.15**	**0.98**	**1.87**	**0.18**
	1	1.00	53	567	0	0	0.09	0.91	1.00	0.00	0.09	0.09	–	1.00	–
RABT	Inf	0.00	0	0	567	53	0.91	0.09	0.00	1.00	–	–	0.91	–	1.00
	3	0.11	22	44	523	31	0.88	0.12	0.42	0.92	0.33	0.33	0.94	5.35	0.63
	**2**	**0.55**	**48**	**290**	**277**	**5**	**0.52**	**0.48**	**0.91**	**0.49**	**0.14**	**0.14**	**0.98**	**1.77**	**0.19**
	1	1.00	53	567	0	0	0.09	0.91	1.00	0.00	0.09	0.09	–	1.00	–

^a^The reported best cutoff is bold.

^b^Note that RABT and ABC start with 1 point, as all the patients included in the study had PTTrauma.

**Figure 3:**
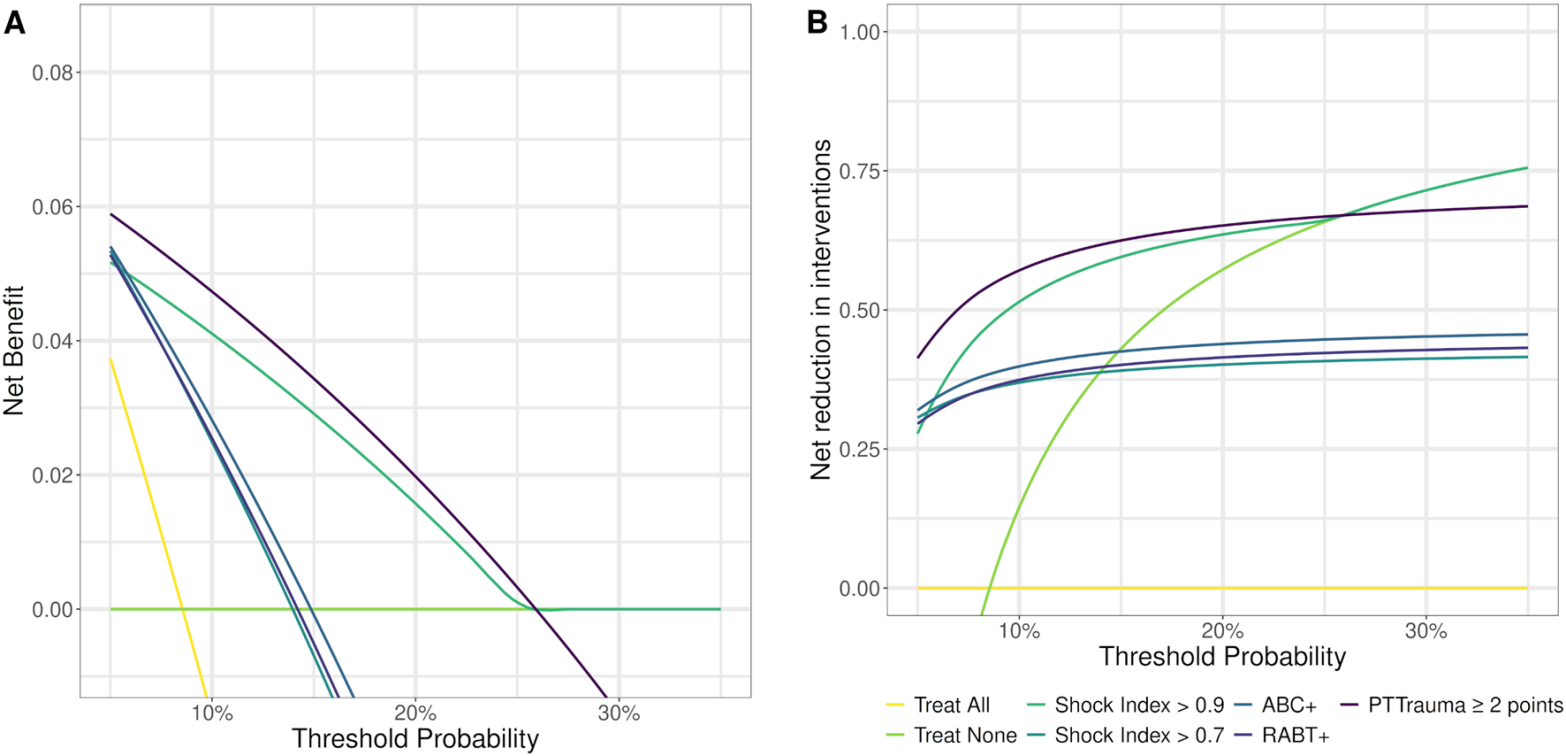
Decision curve analysis curves of each score: PTTrauma, shock index, ABC, and RABT.

The DCA (Fig. 3A) demonstrates the superior net benefit of the PTTrauma score across a wide range of threshold probabilities compared to the SI, ABC, and RABT scores (Fig. 3A). The PTTrauma score consistently shows the highest net benefit, particularly between threshold probabilities of 10% to 30%. Interestingly, the Shock Index, ABC, and RABT scores exhibit similar net benefit curves, indicating that their performance in predicting massive transfusion does not differ significantly from each other. This observation suggests that these scores may have comparable clinical utility in this context, although they are all outperformed by the PTTrauma score.

The PTTrauma score, at a threshold of 2 points, demonstrate the highest number of interventions avoided per 100 patients, particularly between threshold probabilities of 10 to 30%. This finding reinforces the superior clinical utility of the PTTrauma score in identifying patients who require massive transfusion while minimizing unnecessary interventions. The SI, at a cutoff of 0.9, shows the second-highest number of interventions avoided, followed by the ABC and RABT scores (Fig. 3B).

## Discussion

Hemorrhagic shock is a leading cause of preventable death among trauma patients, early recognition and intervention are a crucial strategy for thoracic damage control surgery^42,43^. Various prognostic scores have been developed to predict the likelihood of massive transfusion. However, these scores often underrepresent the trauma epidemiology of low and middle-income countries, and their methodologies typically involve lower frequency penetrating thoracic trauma. In addition, these scores commonly use the classical definition of massive transfusion—more than 10 units of PRBCs in 24 h—which does not account for lesser transfusion needs. The evaluation of SI as a prognostic factor and CAT as a transfusion measure has been underexplored in low and middle-income countries.

In our prospective single-center study of patients with penetrating thorax trauma, the frequency of CAT was 8.5%, which is lower than the 23% reported in the original study deriving CAT but higher than the 7.3% reported in a Korean cohort. The aforementioned studies also showed a higher frequency of CAT among non-surviving patients, which aligns with our findings. However, these studies did not report the penetrating nature of trauma^15,44^.⁠⁠ The higher SI and ISS values observed among non-surviving patients in our study are consistent with previous studies that identified SI as a mortality prognostic factor in penetrating thorax trauma^26^⁠.

The univariate SI AUC was lower (0.81 vs 0.89) than that reported in the Revised Assessment of Bleeding and Transfusion (RABT). However, our multivariate model AUC (0.87 vs 0.89) was similar, demonstrating the importance of specific thoracic trauma variables in predicting transfusion. The RABT is a score derived from the ABC but modified, wherein the transfusion outcome adheres to the traditional definition (more than 10 PRBC in 24 h). It also modifies the cutoff values for heart rate and systolic blood pressure in favor of the SI > 1 cutoff value, and incorporates the FAST ultrasound, the penetrating nature of trauma, and pelvic fracture. The sensitivities in the univariate and multivariate models were similar (84% vs 71%; 79%), as were the specificities (77% vs 80%; 83%)^24,45^⁠. The AUC exhibited a similar trend in both univariate and multivariate models when compared with TASH (0.84 vs. 0.81; 0.87), McLaughlin (0.77 vs. 0.81; 0.87), and ABC (0.86) scores.

McCormick et al. found that adding an SI threshold of ≥ 0.9 to The American College of Surgeons has defined six minimum activation criteria (ACS-6) criteria for trauma team activation yielded a sensitivity of 91.7% and specificity of 64.3% for emergency operative or procedural intervention (EOPI) in a cohort with 15.3% penetrating trauma^46^. Škola et al. showed that prehospital and on-admission SI had excellent negative predictive value (97%) for critically low plasma fibrinogen in a cohort with 2% penetrating trauma, with an AUC of 0.79 for both prehospital and on-admission SI^47^. Bardes et al. demonstrated that prehospital SI was the most significant predictor for blood transfusion and ICU care in patients with blunt torso trauma, but did not report AUC, sensitivity, or specificity values^48^. They emphasized that providers must maintain a high level of clinical suspicion for patients who had an initially elevated SI, as EMS SI was the greatest predictor of injury and need for resources^22^.

In comparison, Kim et al. found that the SI had superior discriminative ability (AUC 0.815) compared to the ABC (AUC 0.797) and RABT (AUC 0.768) scores in predicting in-hospital mortality, ICU admission, and massive transfusion in patients with torso and extremity trauma. However, they did not assess a specific score for penetrating thoracic trauma^49^. Wikström et al. investigated the utility of the SI in predicting survival, functional outcomes, and health status in major trauma patients. They found that adding the shock SI to an existing prediction model resulted in a small but significant improvement in performance, with an increase in AUC from 0.797 to 0.807 for predicting survival to hospital discharge. However, the improvement was marginal for predicting functional outcomes (GOS-E) and health status (EQ-5D-3L) at 6 months post-injury^50^.

Our study extends these findings by focusing specifically on penetrating thoracic trauma and introducing a novel PTTrauma score that incorporates additional clinical variables. The PTTrauma score demonstrated superior performance compared to the SI, ABC, and RABT scores in predicting the need for massive transfusion, as evidenced by the higher net benefit and higher number of interventions avoided across clinically relevant threshold probabilities (10–30%.) in the decision curve analysis.

However, some clinical data may not be recorded in databases, or there could be variables that influence a surgeon's decision to transfuse, which could explain the overlap between the PTTrauma model and the 'treat all' line above the 30% probability threshold^51,52^. These results highlight the importance of developing targeted predictive models for specific injury patterns and mechanisms to optimize clinical utility and guide decision-making in trauma management. A notable benefit of using the SI and the variables incorporated in this model, compared to other scores, lies in the absence of the need for laboratory tests such as lactate or arterial blood gases. In settings with limited resources, this not only reduces costs but also minimizes treatment delays in trauma cases. Additionally, an added advantage of the current model is the utilization of CAT as a transfusion outcome, which allows for the assessment of more specific transfusion needs^22^.

This study has several limitations. First, blood transfusion decisions may be influenced by the clinical judgment of the anesthesiologists, surgeons, and the institutional protocols of the hospital treating the patient. Second, more objective measures for determining transfusion needs, such as serum lactate, base deficit, or hemoglobin levels, were not used in our patient population. Third, this study focused on the hospital phase of trauma care provided to our cohort; many unmeasured factors in the pre-hospital phase could have influenced the initial vital signs. Nonetheless, the study's methodological strengths include an adequate sample size, a cohort design, and the utilization of a high-quality digital blood bank system, which collectively lend more rigor compared to retrospective register-based studies^53,54^. To further validate the prognostic factors and model, multi-institutional studies are needed to assess them from both geographic and temporal perspectives^55^.

In our study, an SI greater than 0.9 exhibited robust discriminative performance in patients with penetrating thoracic trauma, maintaining its efficacy even when incorporated into a multivariate model with relevant clinical variables. Nonetheless, an SI greater than 0.9 by itself may not necessarily act as an immediate trigger for transfusion upon admission. Rather, close hemodynamic monitoring for patients exhibiting this finding may be instrumental in guiding subsequent diagnostic procedures and therapeutic interventions, which may encompass the transfusion of blood components. These insights are congruent with those from various systematic reviews that vouch for the importance of SI in trauma management and investigate its specific role in the context of penetrating thoracic trauma^56^.

In recent advances in treating hemorrhagic shock in trauma, Resuscitative Endovascular Balloon Occlusion of the Aorta (REBOA) has emerged as a pivotal intervention. However, it is explicitly contraindicated in cases of major thoracic hemorrhage or pericardial tamponade^57,58^. Our dataset, considered 'pre-REBOA' information, holds the potential to improve the effectiveness of thoracic trauma reanimation. The SI is highlighted for its valuable predictive capacity in anticipating transfusion needs, especially in the context of major thoracic hemorrhage or pericardial tamponade. When combined with ultrasound or other clinical indicators, this predictive tool promises timely intervention, enhancing overall thoracic trauma resuscitation quality^30^. This data can be utilized as a counterfactual scenario for future causal derivation in a 'post-REBOA' era to evaluate prospective interventions, such as new diagnostic, surgical, or quality improvement programs, the implementation of which may face feasibility challenges within a clinical trial design^59^.

These approaches and the application of advanced statistical methodologies, including machine learning and artificial intelligence, show great promise for enhancing research in trauma care, especially within the pre-hospital phase. Of particular note is the use of information theory to examine the complexity of continuous vital signs. Permutation entropy, recognized for its computational efficiency and resistance to outliers, is an effective tool for quantifying the complexity of time-series data for vital signs^60^.

## Data Availability

The datasets generated and/or analyzed during the current study are not publicly available due to ethical restrictions but are available from the corresponding author on reasonable request.
